# Identification of signatory secondary metabolites during mycoparasitism of *Rhizoctonia solani* by *Stachybotrys elegans*

**DOI:** 10.3389/fmicb.2015.00353

**Published:** 2015-04-29

**Authors:** Rony Chamoun, Konstantinos A. Aliferis, Suha Jabaji

**Affiliations:** Department of Plant Science, McGill UniversitySainte-Anne-de-Bellevue, QC, Canada

**Keywords:** metabolomics, mycoparasitism, mycotoxins, *Rhizoctonia solani*, direct-infusion mass spectrometry

## Abstract

*Stachybotrys elegans* is able to parasitize the fungal plant pathogen *Rhizoctonia solani* AG-3 following a complex and intimate interaction, which, among others, includes the production of cell wall-degrading enzymes, intracellular colonization, and expression of pathogenic process encoding genes. However, information on the metabolome level is non-existent during mycoparasitism. Here, we performed a direct-infusion mass spectrometry (DIMS) metabolomics analysis using an LTQ Orbitrap analyzer in order to detect changes in the profiles of induced secondary metabolites of both partners during this mycoparasitic interaction 4 and 5 days following its establishment. The diketopiperazine(s) (DKPs) cyclo(S-Pro-S-Leu)/cyclo(S-Pro-S-Ile), ethyl 2-phenylacetate, and 3-nitro-4-hydroxybenzoic acid were detected as the primary response of *Rhizoctonia* 4 days following dual-culturing with *Stachybotrys*, whereas only the latter metabolite was up-regulated 1 day later. On the other hand, trichothecenes and atranones were mycoparasite-derived metabolites identified during mycoparasitism 4 and 5 days following dual-culturing. All the above secondary metabolites are known to exhibit bioactivity, including fungitoxicity, and represent key elements that determine the outcome of the interaction being studied. Results could be further exploited in programs for the evaluation of the bioactivity of these metabolites *per se* or their chemical analogs, and/or genetic engineering programs to obtain more efficient mycoparasite strains with improved efficacy and toxicological profiles.

## Introduction

Interactions between microbes encompass antagonistic, mycoparasitic, or competitive outcomes leading to the activation of complex regulatory mechanisms, which are regarded as a major route for the *de novo* biosynthesis of secondary metabolites (Schroeckh et al., 2009; Lorito et al., 2010; Brakhage and Schroeckh, 2011; Brakhage, 2013). Therefore, the study of the fungal secondary metabolites, implicated in such interactions, is expected to provide insights into key factors that determine their outcome.

Mycoparasitism is a complex process when a fungus (mycoparasite) survives by using another fungus (host) as its source of nutrients. This involves a sequence of changes in the metabolism of both partners. Focusing on crop protection, mycoparasitism holds the premise of becoming a valuable component of integrated pest management strategies (IPM) (Viterbo et al., 2007; John et al., 2010). To date, systematic research on mycoparasitism has been mainly performed on *Trichoderma* spp. (Lorito et al., 2010; Druzhinina et al., 2011; Mukherjee et al., 2013). Various other species such as, *Coniothyrium minitans* and *Microsphaeropsis ochracea* (Bitsadze et al., 2014), *Aspergillus aculeatus* (Hu et al., 2013), and *Stachybotrys elegans* (Chamoun et al., 2013), have shown potential as mycoparasites of important plant pathogens. *S. elegans* parasitizes the soil-borne fungal pathogen *Rhizoctonia solani*. During this intimate interaction, *S. elegans* cell wall-degrading enzymes (Taylor et al., 2002; Morissette et al., 2003) and mycoparasitism-associated genes involved in pathogenic processes (Morissette et al., 2008) are expressed. In response to mycoparasitism, transcript levels of a *R. solani* pyridoxal reductase-encoding gene, whose role in reactive oxygen species (ROS) quenching is established, are elevated (Chamoun and Jabaji, 2011).

In contrast to the wide range of applications of metabolomics in plant, animal, and human-related research (Griffin, 2006; Hall, 2006; Spratlin et al., 2009; Aliferis and Jabaji, 2011), microbial metabolomics is still in its infancy. Studies investigating metabolic aspects of microbes have mainly focused on fungal classification (Smedsgaard et al., 2004; Aliferis et al., 2013), metabolic profiling of antagonistic interactions (Tsitsigiannis et al., 2005; Rodriguez Estrada et al., 2011; Combès et al., 2012; Jonkers et al., 2012; Bertrand et al., 2013) or interactions between primary and secondary fungal colonizers of wood (Peiris et al., 2008). Nonetheless, metabolomics has not been yet applied for the study of mycoparasitic interactions.

The main task of the present research is to dissect the undergoing changes in the profile of the secondary bioactive metabolites of both fungal partners during mycoparasitism. This could provide valuable insights into the main factors that determine its outcome. Here, a metabolic profiling strategy was applied performing direct infusion mass spectrometry (DIMS) analysis using a linear trap quadrupole (LTQ) Orbitrap Classic analyzer. Moreover, because metabolite identification represents a bottleneck for fungal metabolomics, (El-Elimat et al., 2013), here it was performed by using a targeted in-house built species-specific metabolic database for *Rhizoctonia* and *Stachybotrys* secondary metabolites. Following dual-culturing, the metabolic profiles of secondary metabolites of *R. solani* and *S. elegans*, were recorded. Such information could be further exploited in crop protection for the production or synthesis of new antifungal agents or for designing selection and genetic engineering programs to obtain more efficient strains of the mycoparasite with improved toxicological profiles.

## Materials and methods

### Chemicals and reagents

All chemicals used for metabolite extraction and sample preparation for DIMS analysis were of the highest commercially available purity. Methanol, ethyl acetate, formic acid, ammonium acetate (Optima® grade), and water (HPLC grade) were purchased from Fisher Scientific Company (Ottawa, ON, Canada).

### Biological material

Starter cultures of the mycoparasite *Stachybotrys elegans* (Pidoplichko) W. Gams (ATCC 18825) and the pathogen *Rhizoctonia solani* AG-3 (ATCC 10183) were revived from pre-colonized oat kernels on 1% potato dextrose agar (PDA; Difco Laboratories, Michigan, USA) and incubated at 24°C for 7 and 5 days, respectively. Induction and collection of *S. elegans* conidia were performed as previously described (Chamoun and Jabaji, 2011).

### Establishment of mycoparasitic interaction

Dual-culturing of *S. elegans* and *R. solani* was conducted in 9 cm Petri plates containing 20 mL of minimal synthetic medium (MSMA) composed (g L^−1^) of: MgSO_4_.7H_2_O, 0.2; K_2_HPO_4_, 0.9; KCl, 0.2; FeSO_4._7H_2_O, 0.002; MnSO_4_, 0.002; ZnSO_4_, 0.002; NaNO_3_, 1.0; biotin, 10 mg; gellan gum, 1% (composed of glucose, glucuronic acid and rhamnose in the molar ratio of 2:1:1) (Phytagel, Sigma, St. Louis, USA).

Agar plugs (8 mm) of a 5-day old *R. solani* culture were grown on MSMA for 48 h and then sprayed with 100 μL of a suspension of *S. elegans* conidia (10^6^ mL^−1^ water) using a Badger 350 air brush and MC-80 mini air compressor calibrated at 1 kg cm^−2^. The control treatments consisted of spraying 100 μL of *S. elegans* conidia on non-inoculated MSMA plates and *R. solani*-inoculated MSMA plates sprayed with sterile distilled water. Additionally, a negative control representing the MSMA medium was used to determine compounds of non-biological origin. All culture plates were incubated at 24°C for 4 or 5 days following dual and pure strain cultivation. These time points were chosen based on *a priori* knowledge to capture the infection and colonization of *R. solani* hyphal cells by *S. elegans* (Chamoun and Jabaji, 2011). Five replications were performed per treatment.

### Optical microscopy

To associate the metabolic changes with the progress of the mycoparasitic process, agar pieces (5 × 5 mm) from interaction zones of dual-culture plates and from pure cultures of both fungal partners were collected in a time course. Sections from interacting zones were stained with lactophenol blue or water and viewed under an optical microscope. Presence of hyphal coils, penetration pegs and intracellular colonization of the pathogen was digitally documented with the Moticam 2300 digital camera (GENEQ Inc. QC, Canada).

### Sampling, quenching, and metabolite extraction

Four plugs (8 mm in diameter × 7 mm in height) were collected from the interaction zones of dual-cultures, pure cultures of each fungal partner after 4 or 5 days of cultivation and from the negative control (MSMA) plates. Plugs were placed in glass autosampler screw thread vials (2 mL, Fisher Scientific, ON, Canada). Quenching was instantly performed by adding twice 2 mL of liquid N_2_, and samples were stored at -80°C until further processing. Extraction was performed as previously described (Aliferis et al., 2014). Briefly, 1 mL of a mixture of methanol-ethyl acetate (50:50, v/v) was added to the vials, followed by sonication for 25 min. Samples were further extracted for 2 h under continuous agitation (250 rpm) at 25°C and filtered through 0.2-μm filters (Millex-FG; Millipore, MA, USA). The volume of samples was adjusted to 1 mL and subsequently divided into two equal portions (0.5 mL) for DIMS analyses in positive (ESI^+^) and negative (ESI^−^) electrospray modes. Finally, extracts were dried using a Labconco CentriVap refrigerated vacuum concentrator equipped with a cold trap (Labconco, MO, USA).

### Direct infusion mass spectrometry (DIMS) and DIMS/MS analysis

For DIMS and DIMS/MS analyses, an LTQ Orbitrap MS Classic (Thermo Scientific, CA, USA) was used acquiring in the ESI^+^ or ESI^−^ modes (Aliferis et al., 2014). All experimental events were controlled by the software Xcalibur v.2.2 (Thermo Scientific). The analyzer was equipped with a heated electrospray ionization probe (HESI-II, Thermo Scientific), a quadrupole linear ion trap, and an Accela pump (Thermo Scientific). For analysis in ESI^+^ and ESI^−^, 100 μL of a mixture of methanol/formic acid (0.2% v/v) (50–50, v/v) or methanol/ammonium acetate (4 mM) was added to the dried samples, respectively. Extracts were then transferred to glass microinserters (150 μL), which were consecutively placed into 2 mL glass autosampler vials. Samples (10 μL) were injected at a flow rate of 10 μL min^−1^ using a 100 μL syringe (Hamilton, NV, USA). Full scan mass spectra were acquired in the range between 50 and 1200 Da at a rate of 0.6 scans/s and a mass resolution of 60,000 at 400 m/z. The source and capillary voltages were set to 3.2 kV and 5.0 V for ESI^+^ and 4.0 kV and −35 V for ESI^−^, respectively. The capillary temperature for both modes was set to 275°C. Sheath gas flow was set to 10 (ESI^+^), and 20 (ESI^−^) whereas no auxiliary and sweep gases were used. For selected samples, MS/MS spectra were recorded using previously described settings (Aliferis et al., 2014).

### Data processing and analysis

Mass spectra were processed using the freely available software MZmine 2 (Pluskal et al., 2010) following the procedures recommended by the developers after optimization of the obtained data. Cumulative spectra were collected between 0.8–1.3 min for ESI^+^ and 0.6–1.1 min for ESI^−^. Metabolic features were detected using the centroid algorithm and the noise level was optimized for each sample. The Fourier transform mass spectrometer (FTMS) shoulder filter was then applied at a mass resolution of 8000 using the Lorentzian extended model function. Chromatogram built, alignment and gap-filling were performed using an *m/z* tolerance (Δppm) <3. Alignment was performed using the “Join aligner” option, whereas gap filling was performed in two steps; first using the “Peak finder option” and then the “Same RT and mz range gap filter” (Pluskal et al., 2010). This procedure accounted for the presence of missing peaks in the matrix as a result of the performance of the peak detection algorithm or possible mistakes in the alignment. Subsequently, the matrices were subjected to filtering by removing rows with more than 50% missing values among the biological replications of the same treatment. Following alignment, metabolic features of non-biological origin corresponding to the negative control samples (MSMA) and also detected in the biological samples were removed and were excluded from further analysis.

The obtained aligned matrix was then exported to Microsoft Excel for further processing. Finally, the matrix composed of identified secondary metabolites detected in ESI^+^ and ESI^−^ was exported to the SIMCA-P+ v.12.0.1 software (Umetrics, MKS Instruments Inc., MA, USA) for multivariate analysis (Aliferis and Jabaji, 2012). The discovery of biomarker-ions was based on partial least squares-discriminant analysis (PLS-DA) regression coefficients (*P* < 0.05). Based on the variability in the model parameters encountered in the different cross-validation cycles, standard errors were calculated with 95% confidence interval using Jack-knifing (Efron and Gong, 1983).

### Metabolite identification and assignment of their origin during mycoparasitism

The identification of metabolites was performed following a biologically-driven approach performing searches against the targeted in-house species-specific metabolic databases for *Rhizoctonia* and *Stachybotrys*. The libraries were constructed acquiring information from the literature and publicly available databases such as, KNApSAcK (http://kanaya.naist.jp/KNApSAcK/) and PubChem (http://pubchem.ncbi.nlm.nih.gov/). Identification of metabolites was based on mass accuracy (<2 ppm) and where available, on isotope and/or MS/MS fragmentation patterns (Supplementary Data Sets 1–4) using data from the databases of METLIN (http://metlin.scripps.edu/index.php) and mzCloud (https://www.mzcloud.org/) and the literature. In addition, the heuristic rules of Kind and Fiehn (2007), which are implemented in the MZmine 2 (Pluskal et al., 2010), were applied. These rules provide a valuable tool for reducing the number of candidate molecular formulae for a given ion. Detection of mass errors was confirmed by Xcalibur v.2.2 (Thermo Scientific).

Additionally, since the majority of the secondary metabolites have unique structures, the assignment of metabolites to the corresponding producing fungus during mycoparasitism was a feasible task at the applied mass resolution.

## Results and discussion

### Morphological and microscopic observations of mycoparasitism

**Figure 1:**
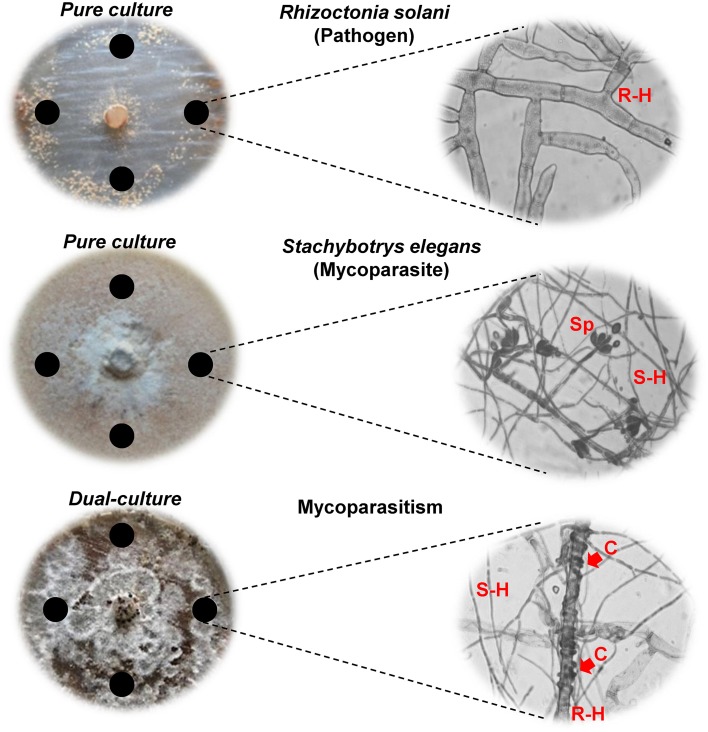
***R. solani* and *S. elegans* in pure and dual-cultures are displayed after 4 days (D4) of growth with corresponding microscope images of hyphae (40X)**. Arrows indicate the formation of coils (C) of *S. elegans* (S-H) on *R. solani* (R-H) hyphae. Black circles indicate the locations of sampling (Sp; spores).

In dual-cultures on Petri plates, *S. elegans* conidia germinated within 24 h, made contact with hyphal cells of *R. solani* and overgrew over the pathogen colonies after 4 days of dual-cultivation (Figure 1). Therefore, 4 and 5 days were selected as the time points to study the induced production, involvement and changement of secondary metabolites during mycoparasitism. Conspicuous accumulation of *S. elegans* aerial hyphae over *R. solani* colonies was observed and accompanied by heavy coiling and formation of infection pegs and intracellular colonization of *R. solani* cells (Figure 1). In the presence of the mycoparasite, the cytoplasm of *R. solani* infected cells appeared disorganized and devoid of granules. *R. solani* pure cultures appeared less pigmented compared to the parasitized cultures (Figure 1), which manifested a change in the color from white to dark brown of underneath medium, corresponding to the biosynthesis and diffusion of fungal metabolites into the growth medium as a result of the interaction (data not shown).

### Metabolite identification and biomarker discovery

The lack of chromatographic separation in DIMS analysis makes the identification of metabolites challenging, even with high mass accuracy (e.g., <2 ppm). In addition, the possible presence of metabolites with identical molecular formulae or isomers makes their absolute identification even more complex. In this context, the identification of metabolites during the mycoparasitic interaction being studied, was based on searches against the two species-specific metabolite libraries for *Stachybotrys* and *Rhizoctonia* (Supplementary Tables 1, 2) for commonly occurred adducts (Supplementary Table 3). Identities were assigned to 36 metabolic features of the obtained metabolite matrix combining results of ESI^+^ and ESI^−^ analyses, 30 of which were unique (single metabolite) (Supplementary Data Set 5). Such approach not only facilitates the robust identification of fungal secondary metabolites, which represents a bottleneck for high-throughput fungal metabolomics, but additionally, it enables the assignment of the origin of the recorded metabolic features in their dual-cultures. The latter is facilitated largely by the unique structures that the identified metabolites of both fungal species have (e.g., none of the metabolites of the two target libraries share the same molecular formula).

**Figure 2:**
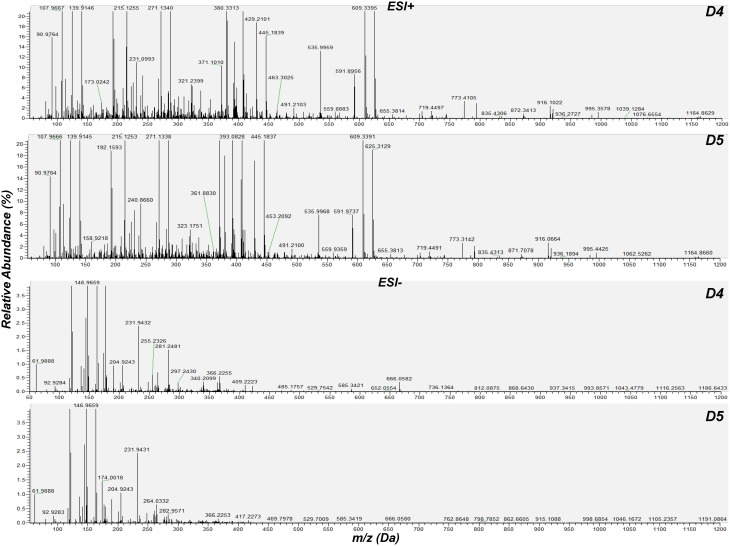
**Cumulative mass spectra of *R. solani*-*S. elegans* dual-cultures 4 (D4) and 5 (D5) days following treatments**. Data were aquired in positive (ESI^+^) and negative (ESI^−^) electrospray modes performing direct infusion analysis in the range 50–1200 Da. The software Xcalibur 2.2 was used for the creation of mass spectra.

**Figure 3:**
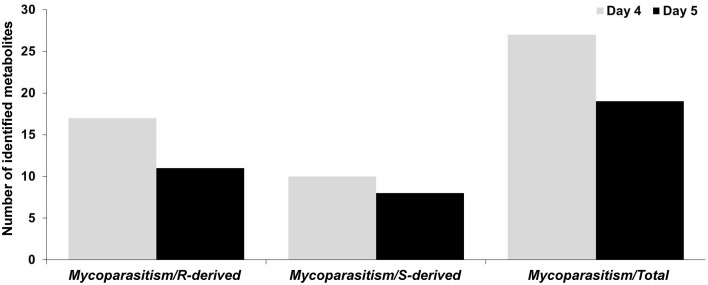
**Number of identified *Rhizoctonia solani* (R)-derived and *Stachybotrys elegans* (S)-derived metabolites during their mycoparasitic interaction 4 and 5 days following dual-culturing**.

For the detection of trends within the obtained matrix and corresponding biomarkers of mycoparasitism, the metabolic profiles of *R. solani*-*S. elegans* dual-cultures were compared to those of pure cultures for both time points (Figure 2 and Supplementary Figures 1–4) applying multivariate analysis (MVA). Initially, application of the unsupervised principal component analysis (PCA), revealed a tight clustering between the biological replications of the same treatment in the corresponding PC1/PC2 score plots and the absence of outliers (*P* < 0.05) (Supplementary Figure 5). This is indicative of the robustness of the applied bio-analytical protocol, data processing, instrument's performance and of the substantial differences between the metabolic profiles of *Rhizoctonia-Stachybotrys* dual-cultures and their corresponding pure cultures at both time points. Additionally, the number of identified *Rhizoctonia*-derived metabolites was substantially higher than that of *Stachybotrys*-derived ones for both time points (Figure 3). Plausibly this is due to the fact that *Rhizoctonia* was established in the media prior to treatments, which gave it more time to synthesize and release metabolites.

**Figure 4:**
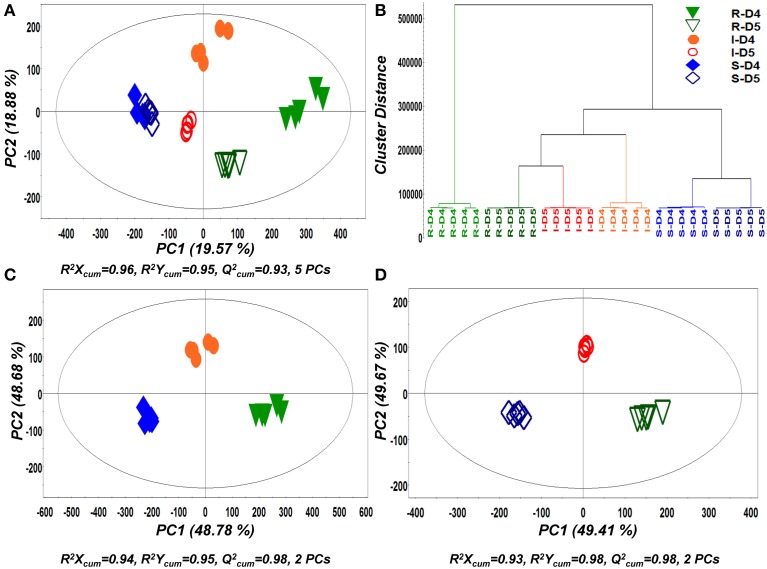
**Partial least squares-discriminant analyses (PLS-DA) PC1/PC2 score plots of identified secondary metabolite profiles of *Rhizoctonia solani* (R), *Stachybotrys elegans* (S), and their dual-cultures (I), 4 (D4) and 5 (D5) days following inoculation (A) and corresponding PLS-dendrogram perfoming hierarchical cluster analysis (HCA) (B)**. PLS-DA PC1/PC2 score plots for D4 **(C)** and D5 **(D)** are also displayed. In the score plots, the ellipse represents the Hotelling T^2^ with 95% confidence interval. Five (5) biological replications were performed per treatment [*Q^2^(cum)*; cumulative fraction of the total variation of the *X*'s that can be predicted by the extracted components, *R^2^X* and *R^2^Y*; the fraction of the sum of squares of all *X*'s and *Y*'s explained by the current component, respectively].

In a second step of MVA, PLS-DA and hierarchical clustering were applied for the discovery of trends within treatments (Figure 4). Similarly to PCA, both analyses showed a very strong discrimination between the recorded metabolic profiles of pure and dual-cultures and tight clustering between biological replications. As an indication of the dynamics of its biosynthetic activity, the metabolite profiles of *Rhizoctonia* grown in pure and in dual-cultures were substantially different at both time points.

It is noteworthy, that for metabolomics, the absence of a metabolite from a treatment in the matrix means either no detection (e.g., below the limits of detection) or elimination following filtering (missing values >50%). The latter could be attributed either to the variation in the biological samples or signals with intensity near the limits of detection of the instrument.

### Mycoparasitism by *Stachybotrys elegans* affects significantly the biosynthesis of *Rhizoctonia solani* secondary metabolites

**Figure 5:**
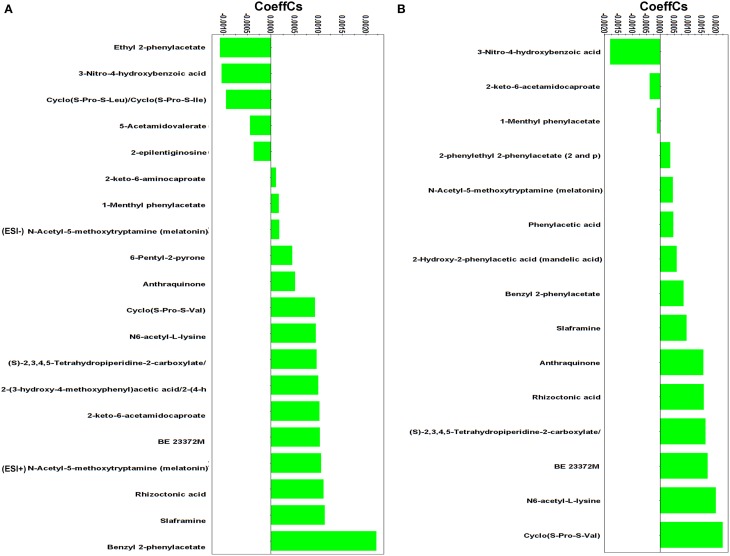
**Partial least squares (PLS) coefficient plots for the comparison between *Rhizoctonia solani* pure cultures and its dual-cultures with *Stachybotrys elegans* at D4 (A) and D5 (B) days with values of scaled and centered PLS regression coefficients (CoeffCS)**. Negative values of coefficients denote metabolites with higher concentration during mycoparasitism whereas positive values correspond to those with higher concentration in *Rhizoctonia* monocultures. Values < −0.0005 and > +0.0005 were considered significant (ESI^+^; positive electrospray mode, ESI^−^; negative electrospray mode).

Results revealed the substantial impact of *Stachybotrys* mycoparasitic activity on *Rhizoctonia's* metabolism (Figures 4, 5). The biosynthesis of the vast majority of the identified *Rhizoctonia*-derived metabolites were significantly down-regulated, whereas only a handful was up-regulated or remained unaffected in response to mycoparasitism. This is indicative of the general disturbance of the pathogen's metabolism in response to the invasion of the mycoparasite, which plausibly represents the evidence for the outcome of such interaction.

The diketopiperazines (DKPs) cyclo(S-Pro-S-Leu)/cyclo(S-Pro-S-Ile), ethyl 2-phenylacetate, and 3-nitro-4-hydroxybenzoic acid (Supplementary Figure 6) were induced in *Rhizoctonia* 4 days following dual-cultivation with *Stachybotrys*, whereas only the latter was found to be up-regulated 1 day later.

DKPs exhibit antifungal and antibacterial properties, and inhibit the biosynthesis of mycotoxins (Martins and Carvalho, 2007; Huang et al., 2010; Borthwick, 2012). They have been isolated from fungal species such as, *Aspergillus* spp. (Li et al., 2004; Wang et al., 2008), *Alternaria* spp. (Musetti et al., 2007), *Fusarium oxysporum* (Trigos et al., 1995), and *R. solani* (Pedras et al., 2005). In the latter study, the DKPs cyclo(S-Pro-S-Leu)/cyclo(S-Pro-S-Ile) were isolated from *R. solani* cultures, which is in accordance with our data. Cyclo(S-Pro-S-Leu)/cyclo(S-Pro-S-Ile) do not exhibit phytotoxicity (Pedras et al., 2005), however, based on the resemblance of their structure with other DKPs with established antimicrobial and mycotoxin inhibitory action, it is plausible to suggest a role of cyclo(S-Pro-S-Leu)/cyclo(S-Pro-S-Ile) in the defense mechanism of *Rhizoctonia* against the stress imposed by the invasive mycelia of *Stachybotrys*, a known producer of mycotoxins (Deng et al., 2003). In contrast, the biosynthesis of cyclo(S-Pro-S-Val) (Figure 5) was suppressed in the presence of the mycoparasite at both time points.

Phenylacetic acid (PAA) and its derivatives are the first studied bioactive metabolites of *Rhizoctonia* (Aoki et al., 1963). These metabolites share a functional phenyl group and a carboxylic acid (Supplementary Figure 6), and are known not only for their phytotoxicity but also for their antimicrobial activities (Hwang et al., 2001; Mao et al., 2006; Ding et al., 2008; de Lima Mendonça et al., 2009). Interestingly, here, the biosynthesis of PAA, phenylethyl-2-phenylacetate, and mandelic acid was suppressed in the presence of the mycoparasite; whereas ethyl 2-phenylacetate and 1-menthyl phenylacetate were the most induced metabolites in response to *Stachybotrys* attack (Supplementary Figure 6). A major response of *Rhizoctonia* to mycoparasitism was the increased biosynthesis of 3-nitro-4-hydroxybenzoic acid at both time points. Information in the literature supporting the antimicrobial activity of benzoic acid and its derivatives against economically important plant pathogens is established (Sopheareth et al., 2013). Its mode of action is attributed to lowering the intracellular pH of fungal cells leading to inhibition of glycolysis (Krebs et al., 1983). Whether 3-nitro-4-hydroxybenzoic acid has the same role during the mycoparasitic process of *R. solani* by *S. elegans* requires further study.

The success of *Stachybotrys* to overcome the defense mechanisms of *Rhizoctonia* and parasitize it is indirectly linked to the suppression of the biosynthesis of the majority of its metabolites with well-established bioactivity. Among these, melatonin is an antioxidant and free radical scavenger in many organisms, including fungi (Hardeland et al., 2006; Tamura et al., 2012). Interestingly, N6-acetyl-L-lysine and (S)-2,3,4,5-tetrahydropiperidine-2-carboxylate that belong to the lysine degradation pathway were detected in lower amounts during mycoparasitism compared to pure cultures. Both metabolites control the synthesis of glutamate which contributes to the synthesis of the stress-related molecules *γ*-aminobutyric acid, polyamines, and nitric oxide (Galili, 2002).

Fungal species are also sources of pigments (Gessler et al., 2013). Among them are octaketide pigments with structure based on the anthraquinone skeleton (Velíšek and Cejpek, 2011). Here, a decrease in anthraquinone was observed in the dual-cultures compared to the pure cultures 4 days following treatment, whereas the metabolite was not detected in the dual-cultures 1 day later. Bioassay studies performed on several anthraquinones derivatives that were isolated from various fungi have highlighted their antibacterial, antiparasitic, antiviral and fungicidal activities (Kanokmedhakul et al., 2002; Srinivas et al., 2007; Zhou et al., 2014). The metabolite slaframine, known to be produced by *R. leguminicola*, the causal pathogen of the black patch disease of red clover (Li et al., 2012), has been detected also in decreased amount in pure cultures compared to dual-cultures at both time points. Slaframine is an indolizidine alkaloid responsible for locoism and leads to economical losses in animals (Croom et al., 1995).

The benzophenone derivative rhizoctonic acid was present only in pure cultures and suppressed during mycoparasitism. In addition to its isolation from *R. solani* (Ma et al., 2004), it has been isolated from endophytic fungi such as, *Guignardia* and *Penicillium* sp. and has been reported to exhibit antimicrobial activity against human pathogens (Ma et al., 2004; Wang et al., 2010).

### Secondary metabolites of *Stachybotrys elegans* involved in mycoparasitism

**Figure 6:**
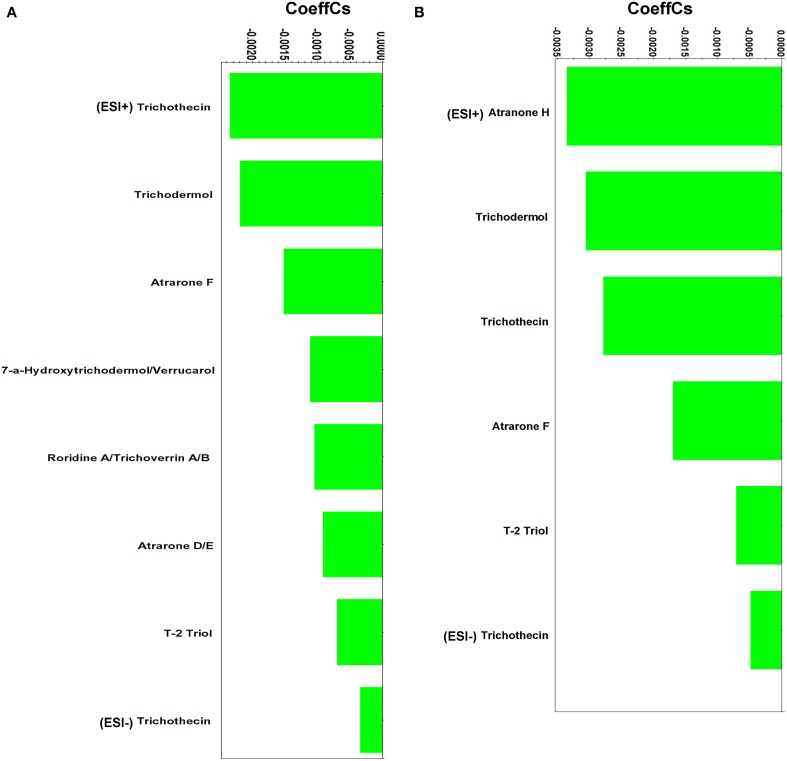
**Partial least squares (PLS) coefficient plots for *Stachybotrys elegans*-derived metabolites that were identified during mycoparasitism with values of scaled and centered PLS regression coefficients (CoeffCS) (ESI^+^; positive electrospray mode, ESI^−^; negative electrospray mode)**.

*Stachybotrys* genus includes diverse species having the ability to produce a wide range of bioactive secondary metabolites (Deng et al., 2003). Several mycotoxins of *Stachybotrys* were detected during *Rhizoctonia*'s mycoparasitism 4 and 5 days following dual-culturing (Figure 6 and Supplementary Data Set 5), which probably suggesting their involvement in the mycoparasitism process. The production of mycotoxins by the genus *Stachybotrys* as well as other unrelated fungi such as, *Fusarium*, *Trichoderma*, *Trichothecium*, *Verticimonosporium*, and *Cephalosporium* is well documented (Bräse et al., 2009; McCormick et al., 2011; Kramer and Abraham, 2012).

The majority of the identified metabolites belongs to trichothecenes, a well-studied class of sesquiterpenes (Rocha et al., 2005; McCormick et al., 2011). They are able to passively move across cell membranes (McCormick et al., 2011) and bind to ribosomes and trigger mitogen-activated protein kinases (MAPKs) (Pestka et al., 2004). Their bioactivity is mainly attributed to the epoxide that they contain (C12-C13) (Sudakin, 2003) (Supplementary Figure 6).

The identified trichothecenes are simple (e.g., trichothecin andtrichodermol), with the exception of the macrocyclicroridin A-trichoverrin A/B (Supplementary Data Set 5). Studies performed on human and plant cells have revealed that trichothecenes act by inhibiting the synthesis of nucleic acids and protein synthesis (Rocha et al., 2005; McCormick et al., 2011). Additionally, trichothecenes have been reported to generate hydrogen peroxide, alter cell division and membrane function (Shifrin and Anderson, 1999; Nishiuchi et al., 2006; Yazar and Omurtag, 2008). Reports on trichothecenes' activity against plant pathogens are less common (Ayer and Miao, 1993). Trichodermol produced by *Stachybotrys cylindrospoa* exhibited strong activity against the blue stain fungus *Ophiostoma crassivaginatum* in confrontation assays (Hiratsuka et al., 1994). In a similar analogy to their documented bioactivity on human and plant cells, we hypothesize that the presence of trichothecenes in dual-cultures is triggered by the pathogen and results in the alteration of its metabolism and ultimately its growth and development.

In addition to trichothecenes, the *Stachybortys*-produced atranones D/E, F and H, were identified during mycoparasitism, with the latter being present only during mycoparasitism and not in pure cultures (Supplementary Data Set 5). This indicates its *de novo* or substantially increased biosynthesis during mycoparasitism. This toxin is an analog of atranones A, B, and I (Hinkley et al., 2003) and a precursor of atranone J (Jarvis, 2003). Atranones are diterpenoids with unique structures (Supplementary Figure 6), produced by species such as, *Stachybotrys* spp. and *Myrothecium verrucaria* (Bräse et al., 2009). However, in contrast to trichothecenes, atranones do not exhibit significant bioactivity (Jarvis, 2003).

Finally, a small number of identified *Stachybotrys* metabolites were detected only in pure cultures (Supplementary Figure 7). Among these, spirodihydrobenzofuranlactam 4 (Deng et al., 2003) act as protein synthesis inhibitors and protein antagonists (Roggo et al., 1996); the spirocyclic drimane stachybotrylactone exhibit antiplasmodial activity (Wang et al., 2014); and the sesquiterpenoid trichothecolone exhibits cytotoxic activity (Wang et al., 2015). This finding plausibly indicates their decreased biosynthesis and thus, minor importance for mycoparasitism. This could be attributed either to a “preference” for the biosynthesis of other bioactive metabolites or inhibition of their biosynthesis as a result of the action of *Rhizoctonia*-derived metabolites, and needs further investigation.

## Conclusion

An original DIMS metabolomics approach was developed for the monitoring of the production of secondary bioactive metabolites in interaction zones of hyphal mycelia formed between a mycoparasite and a fungal pathogen during active mycoparasitism. In these zones both partners are subjected to intense stress leading to the induction of secondary bioactive metabolites for attack and/or defense. The majority of the antimicrobial *R. solani*-derived metabolites were down-regulated in dual-cultures possibly due to the direct effect of the mycoparasite on host's metabolism or because they were produced in trace amounts. Alternatively, *S. elegans* mycotoxins known as trichothecenes were up-regulated during mycoparasitism. To the best of our knowledge, this is the first report on the involvement of trichothecenes in the active process of mycoparasitism. Results could be further exploited in programs for the evaluation of the bioactivity of these metabolites *per se*, or their structures as chemical analogs and/or genetic engineering programs to obtain more efficient mycoparasite strains with improved efficacy and toxicological profiles. Experiments are underway to isolate the most induced metabolites from each fungal partner and test their bioactivity against each other.

## Author contributions

RC, KA, and SJ conceived, designed and executed the experiments. RC and KA analyzed the data. RC, KA, and SJ contributed to the writing of the manuscript.

### Conflict of interest statement

The authors declare that the research was conducted in the absence of any commercial or financial relationships that could be construed as a potential conflict of interest.
