# Supplementary material for: Identification of signatory secondary metabolites during mycoparasitism of Rhizoctonia solani by Stachybotrys elegans
Source: Front Microbiol. 2015 Apr 29;6:353. doi: 10.3389/fmicb.2015.00353 (PMC4413796; doi:10.3389/fmicb.2015.00353)
Supplement: Supplementary file 1 [file Table1.DOCX]

***Supplementary Material***

**Involvement of diverse secondary metabolites in the interaction between the plant pathogen *Rhizoctonia solani* and the mycoparasite *Stachybotrys elegans***

Rony Chamoun, Konstantinos A. Aliferis, Suha Jabaji*

Department of Plant Science, McGill University, Sainte-Anne-de-Bellevue, Quebec, Canada

*** Correspondence:**

Dr. Suha Jabaji

McGill University

Department of Plant Science

21111 Lakeshore Rd., Sainte-Anne-de-Bellevue, Quebec, H9X 3V9, Canada

E-mail:[suha.jabaji@mcgill.ca](mailto:suha.jabaji@mcgill.ca)

Tel: +1 514-398-7561

1. **Supplementary Data Sets**

**Supplementary Data Set 1** **Chromatograms of *Rhizoctonia solani*-*Stachybotrys elegans*** **dual-cultures after four (D4)** **days of growth** **acquired performing direct infusion mass spectrometry (DIMS) analysis in the positive** **electrospray mode (ESI^+^) using an LTQ Orbitrap Classic analyzer.** For details on analysis consult the section of Materials and Methods.

**Supplementary Data Set 2** **Chromatograms of *Rhizoctonia solani*-*Stachybotrys elegans* dual-cultures after five (D5) days of growth acquired performing direct infusion mass spectrometry (DIMS) analysis in the positive electrospray mode (ESI^+^) using an LTQ Orbitrap Classic analyzer.** For details on analysis consult the section of Materials and Methods.

**Supplementary Data Set 3 Chromatograms of *Rhizoctonia solani*-*Stachybotrys elegans* dual-cultures after four (D4) days of growth acquired performing direct infusion mass spectrometry (DIMS) analysis in the negative electrospray mode (ESI^-^) using an LTQ Orbitrap Classic analyzer.** For details on analysis consult the section of Materials and Methods.

**Supplementary Data Set 4 Chromatograms of *Rhizoctonia solani*-*Stachybotrys elegans* dual-cultures five (D5) days of growth acquired performing direct infusion mass spectrometry (DIMS) analysis in the negative electrospray mode (ESI^-^) using an LTQ Orbitrap Classic analyzer.** For details on analysis consult the section of Materials and Methods.

**Supplementary Data Set 5 Matrix that was subjected to multivariate analysis using the software SIMCA-P+ v.12.0.1.** The matrix was obtained following pre-processing of DIMS chromatograms using the software MZmine 2. Identified *Rhizoctonia solani*-derived and *Stachybotrys elegans*-derived metabolites with corresponding values of absolute ion intensities are displayed within an average mass error (Δppm) <2. Analyses were performed applying direct infusion mass spectrometry (DIMS) using an LTQ Orbitrap Classic analyzer in positive (ESI^+^) and negative (ESI^-^) electrospray modes of pure cultures of *S. elegans* (S) and *R. solani* (R) and dual-cultures (I) after four (D4) and five (D5) days of growth. Biomarkers were selected based on their PLS-DA regression coefficients (*P < 0.05*). Putative molecular formulae were assigned after searches against an in-house species-specific metabolic database within a mass error (Δppm) <2.

1. **Supplementary Figures and Tables**
   1. **Supplementary Tables**

**Supplementary Table 1 Target in-house-built library for *Rhizoctonia solani*** **secondary metabolites used in the identification of** ***Rhizoctonia*-derived metabolic features**

| **Monoisotopic Mass (Da)** | **Metabolite** | **Molecular Formula** |
| --- | --- | --- |
| 102.1157 | Cadaverine | C_5_H_16_N_2_ |
| 110.0368 | Benzenediol | C_6_H_6_O_2_ |
| 112.0160 | 2-Furoate (Pyromucic acid) | C_5_H_4_O_3_ |
| 117.0790 | 5-Aminopentanoate | C_5_H_11_NO_2_ |
| 127.0633 | (S)-2,3,4,5-Tetrahydropiperidine-2-carboxylate | C_6_H_8_NO_2_ |
| 127.0633 | Δ1-Piperideine-2-carboxylate | C_6_H_8_NO_2_ |
| 136.0524 | Phenylacetic acid | C_8_H_8_O_2_ |
| 139.9875 | Acetylphosphate | C_2_H_3_O_5_P |
| 145.0739 | (S)-2-Amino-6-oxohexanoate | C_6_H_11_NO_3_ |
| 145.0739 | (S)-5-Amino-3-oxohexanoate | C_6_H_11_NO_3_ |
| 145.0739 | 2-Keto-6-aminocaproate | C_6_H_11_NO_3_ |
| 150.0317 | 2-Oxo-2-phenylacetic acid | C_8_H_6_O_3_ |
| 152.0473 | 2-H-2-Phenylacetic acid (mandelic acid) | C_8_H_8_O_3_ |
| 157.1103 | 2-Epilentiginosine | C_8_H_15_NO_2_ |
| 159.0895 | 5-Acetamidovalerate | C_7_H_12_NO_3_ |
| 160.0372 | 2-Oxoadipate | C_6_H_6_O_5_ |
| 161.0688 | L-2-Aminoadipate | C_6_H_10_NO_4_ |
| 164.0837 | Ethyl 2-phenylacetate | C_10_H_12_O_2_ |
| 166.0994 | 6-Pentyl-2-pyrone | C_10_H_14_O_2_ |
| 168.0423 | 2-(3,4-dihydroxyphenyl)acetic acid | C_8_H_8_O_4_ |
| 173.1052 | Swainsonine | C_8_H_15_NO_3_ |
| 182.0579 | 2-(3-hydroxy-4-methoxyphenyl)acetic acid | C_9_H_10_O_4_ |
| 182.0579 | 2-(4-hydroxy-3-methoxyphenyl)acetic acid | C_9_H_10_O_4_ |
| 183.0168 | 3-Nitro-4-hydroxybenzoic acid | C_7_H_5_NO_5_ |
| 187.0845 | 2-Keto-6-acetamidocaproate | C_8_H_12_NO_4_ |
| 188.1161 | N6-acetyl-L-lysine | C_8_H_16_N_2_O_3_ |
| 196.1212 | Cyclo(S-Pro-S-Val) | C_10_H_16_N_2_O_2_ |
| 198.1368 | Slaframine | C_10_H_18_N_2_O_2_ |
| 202.1106 | Nb-acetyltryptamine | C_12_H_14_N_2_O |
| 208.0524 | Anthraquinone | C_14_H_8_O_2_ |
| 210.1368 | Cyclo(S-Pro-S-Ile) | C_11_H_18_N_2_O_2_ |
| 210.1368 | Cyclo(S-Pro-S-Leu) | C_11_H_18_N_2_O_2_ |
| 226.0994 | Benzyl 2-phenylacetate | C_15_H_14_O_2_ |
| 232.1212 | N-Acetyl-5-methoxytryptamine (Melatonin) | C_13_H_16_N_2_O_2_ |
| 240.1150 | 2-Phenylethyl 2-phenylacetate (2 and p) | C_16_H_16_O_2_ |
| 274.1933 | 1-Menthyl phenylacetate | C_18_H_26_O_2_ |
| 312.0634 | BE 23372M | C_17_H_12_O_6_ |
| 332.0896 | Rhizoctonic acid | C_17_H_16_O_7_ |
| 346.1053 | Monomethylsulochrin | C_18_H_18_O_7_ |

**Supplementary Table 2 Target in-house-built library for *Stachybotrys elegans* secondary metabolites used in the identification of** ***Stachybotrys*-derived metabolic features**

| **Monoisotopic Mass (Da)** | **Metabolite** | **Molecular Formula** |
| --- | --- | --- |
| 250.1569 | Trichodermol | C_15_H_22_O_3_ |
| 264.1362 | Trichothecolone | C_15_H_20_O_4_ |
| 266.1518 | 7-a-Hydroxytrichodermol | C_15_H_22_O_4_ |
| 266.1518 | Verrucarol | C_15_H_22_O_4_ |
| 282.1467 | Scirpentriol | C_15_H_22_O_5_ |
| 324.1573 | 15-Acetoxyscirpenol | C_17_H_24_O_6_ |
| 326.0395 | Gliotoxin | C_13_H_14_N_2_O_4_S_2_ |
| 332.1624 | Trichothecin | C_19_H_24_O_5_ |
| 338.1366 | 15-O-Acetyl-4-deoxynivalenol | C_17_H_22_O_7_ |
| 350.1729 | 4,15-Diacetylverrucarol | C_19_H_26_O_6_ |
| 354.1315 | Fusarenone X | C_17_H_22_O_8_ |
| 366.1679 | Diacetoxyscirpenol | C_19_H_26_O_7_ |
| 382.1628 | Neosolaniol | C_19_H_26_O_8_ |
| 382.1992 | T-2 Triol | C_20_H_30_O_7_ |
| 385.2253 | Spirodihydrobenzofuranlactam 1 | C_23_H_31_NO_4_ |
| 385.2253 | Stachybotrylactam/Stachyflin | C_23_H_31_NO_4_ |
| 386.2093 | Stachybotrylactone | C_23_H_35_O_5_ |
| 386.2457 | Atrarone D/E | C_24_H_34_O_4_ |
| 388.2250 | L671 776 | C_23_H_32_O_5_ |
| 401.2202 | Stachybotrin A | C_23_H_31_NO_5_ |
| 404.2199 | Atranone J | C_23_H_32_O_6_ |
| 406.2719 | Dolabellanediterpene | C_24_H_38_O_5_ |
| 408.1784 | 3a-Acetyldiacetoxyscirpenol | C_21_H_28_O_8_ |
| 416.2199 | Atrarone A | C_24_H_32_O_6_ |
| 416.2199 | Atranone C | C_24_H_32_O_6_ |
| 420.2150 | Trichoverrol A/B | C_23_H_32_O_7_ |
| 424.2097 | HT-2 Toxin | C_22_H_32_O_8_ |
| 429.2515 | Spirodihydrobenzofuranlactam 2 | C_25_H_35_NO_5_ |
| 432.2148 | Atrarone F | C_24_H_32_O_7_ |
| 446.2305 | Atrarone B | C_25_H_34_O_7_ |
| 448.2097 | Atranone H | C_24_H_32_O_8_ |
| 462.2254 | Atranone G | C_25_H_34_O_8_ |
| 466.2202 | Insariotoxin (T-2 toxin) | C_24_H_34_O_9_ |
| 484.2097 | iso-Verrucarin J | C_27_H_32_O_8_ |
| 484.2097 | Verrucarin J | C_27_H_32_O_8_ |
| 485.2777 | Spirodihydrobenzofuranlactam 3/  Staplabin | C_28_H_39_NO_6_ |
| 500.2046 | Verrucarin B | C_27_H_32_O_9_ |
| 502.2203 | Verrucarin A | C_27_H_34_O_9_ |
| 508.2308 | Acetyl-T-2 toxin | C_26_H_36_O_10_ |
| 512.2410 | Iso-roridine H | C_29_H_36_O_8_ |
| 512.2410 | Roridine H | C_29_H_36_O_8_ |
| 514.2567 | Epi-roridine E | C_29_H_38_O_8_ |
| 514.2567 | Iso-roridine E | C_29_H_38_O_8_ |
| 514.2567 | Roridine E | C_29_H_38_O_8_ |
| 515.2519 | Spirodihydrobenzofuranlactam 4 | C_28_H_37_NO_8_ |
| 528.2359 | Satratoxin H | C_29_H_36_O_9_ |
| 530.2516 | Hydroxy-Roridin E | C_29_H_38_O_9_ |
| 530.2516 | Roridin L2 | C_29_H_38_O_9_ |
| 532.2672 | Roridine A | C_29_H_40_O_9_ |
| 532.2672 | Trichoverrin A/B | C_29_H_40_O_9_ |
| 542.2152 | Iso-satratoxin F | C_29_H_34_O_10_ |
| 542.2152 | Satratoxin F | C_29_H_34_O_10_ |
| 544.2309 | Satratoxin G | C_29_H_36_O_10_ |
| 882.5030 | Stachybocin A | C_52_H_70_N_2_O_10_ |
| 914.4929 | Stachybocin D | C_52_H_70_N_2_O_12_ |

**Supplementary Table 3 Adducts for which searches against the species-specific databases of *Rhizoctonia solani* and *Stachybotrys elegans* were performed** **within a mass error (Δppm) <2**

| **Electrospray ionization mode** | **Adducts** |
| --- | --- |
| ESI^+^ | [M+H]^+^, [M+K]^+^, [M+Na]^+^, [M+NH_4_]^+^, [2M+H]^+^, [3M+H]^+^, [M+H_2_O+H]^+^, [M+Na+2H]^+^, [M+H+2Na]^+^, [M- H_2_O+H]^+^, [M+CH_3_OH+H]^+^, [M+CH_3_CN+H]^+^ |
| ESI^-^ | [M-H]^-^, [M+H_2_O-H]^-^, [M+Na-2H]^-^, [M+2H_2_O-H]^-^, [M+K-2H]^-^, [M+CH_3_CN-H]^-^, [M+HCOOH-H]^-^, [M+NaCOOH-H]^-^, [M+KCOOH-H]^-^, [M+2COOH-H]^-^, [M+H_2_SO_4_-H]^-^, [2M-H]^-^, [3M-H]^-^ |

- 1. **Supplementary Figures**

**
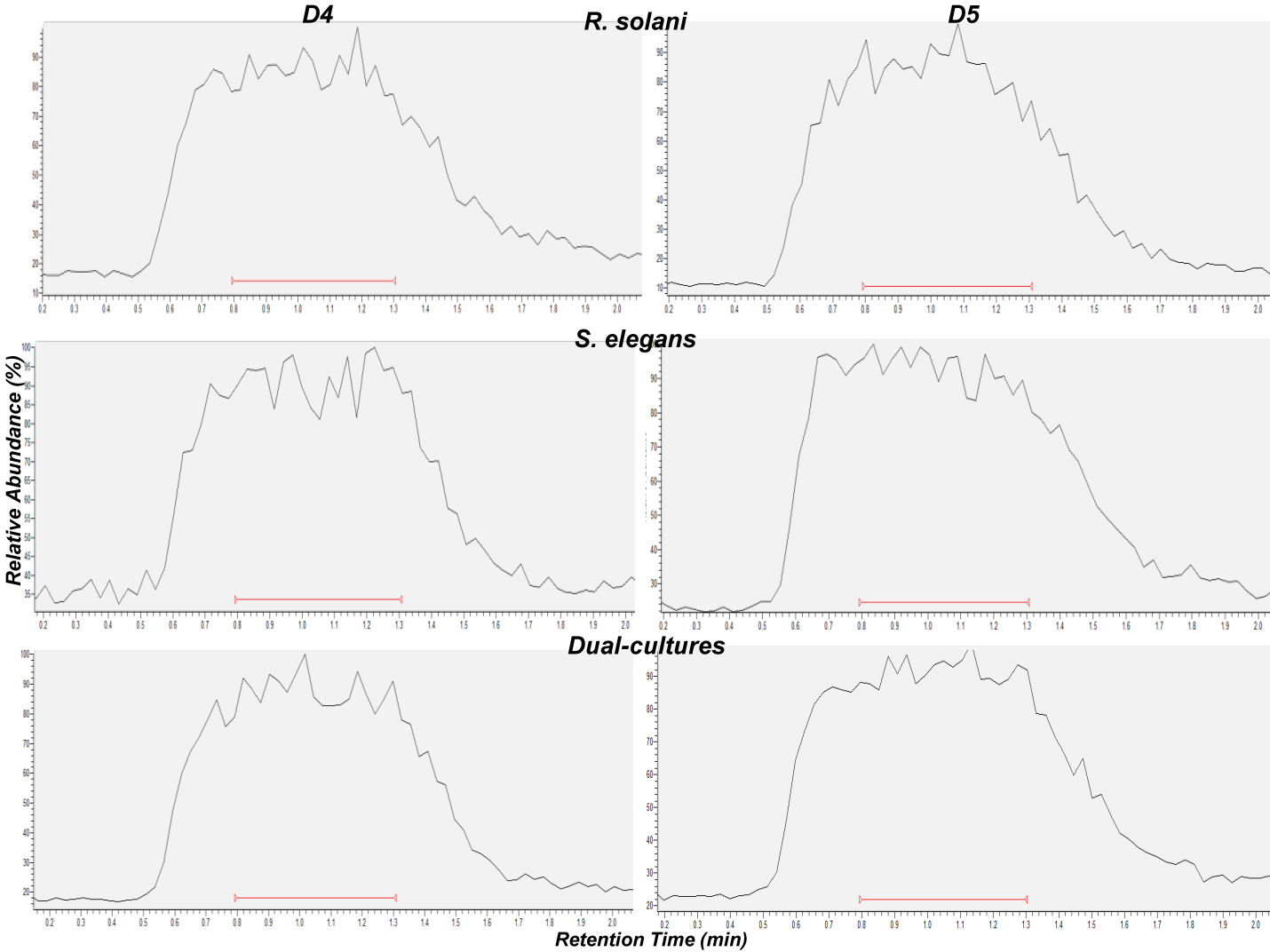
**

**Supplementary Figure 1 Representative total ion chromatograms (TIC) of extracts of *Rhizoctonia solani*, *Stachybotrys elegans* and their dual-cultures four (D4) and five (D5) days following treatments aquired in the positive electrospray mode (ESI^+^) performing direct infusion mass spectrometry (DIMS) analysis** **using an LTQ Orbitrap Classic analyzer.** The area between the red arrow was used to obtain the cumulative mass spectra.

**
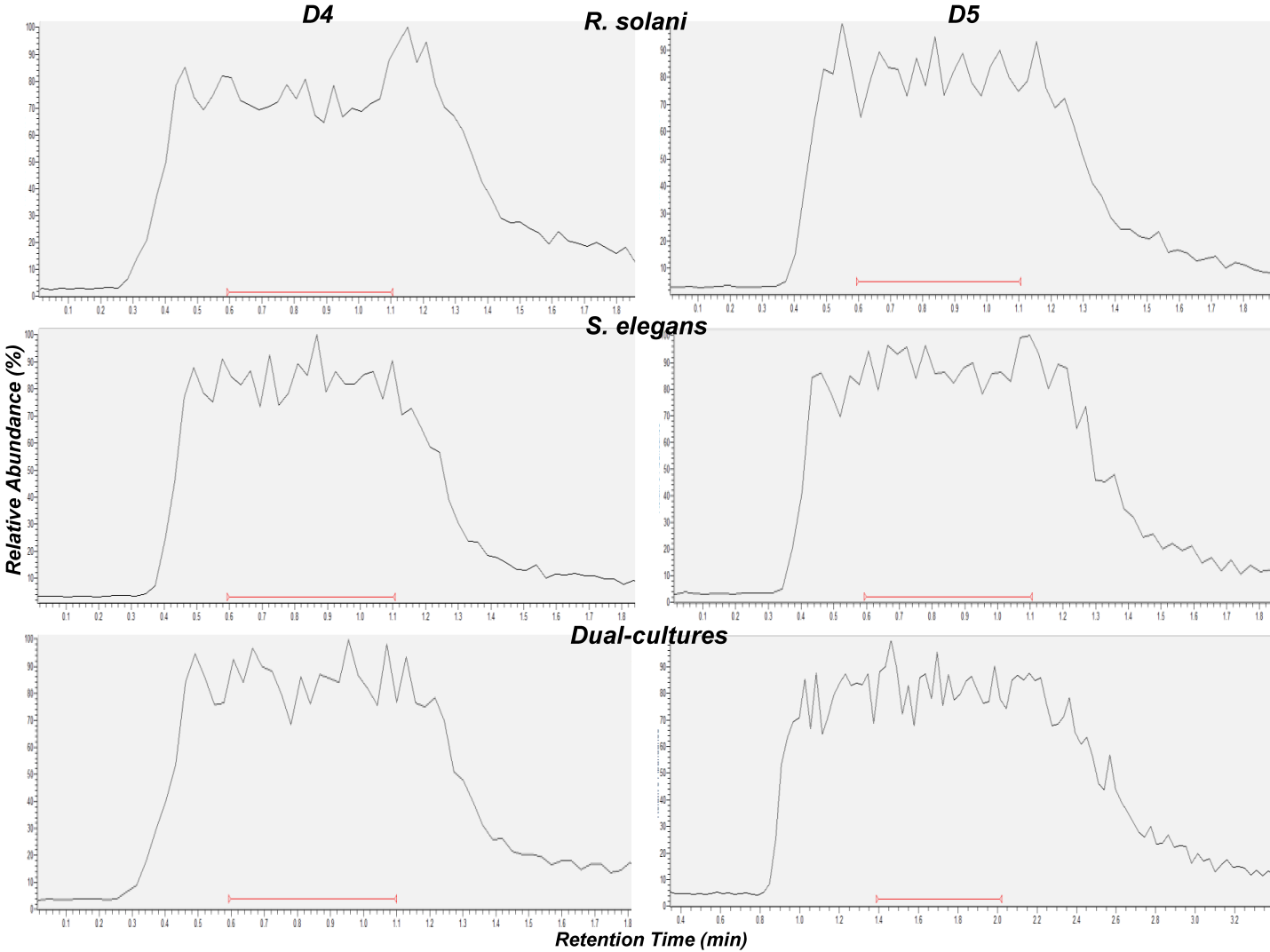
**

**Supplementary Figure 2 Representative total ion chromatograms (TIC) of extracts of *Rhizoctonia solani*, *Stachybotrys elegans* and dual-cultures four (D4) and five (D5) days following treatments aquired in the negative electrospray mode (ESI^-^) performing direct infusion mass spectrometry (DIMS) analysis using an LTQ Orbitrap Classic analyzer.** The area between the red arrow was used to obtain the cumulative mass spectra.


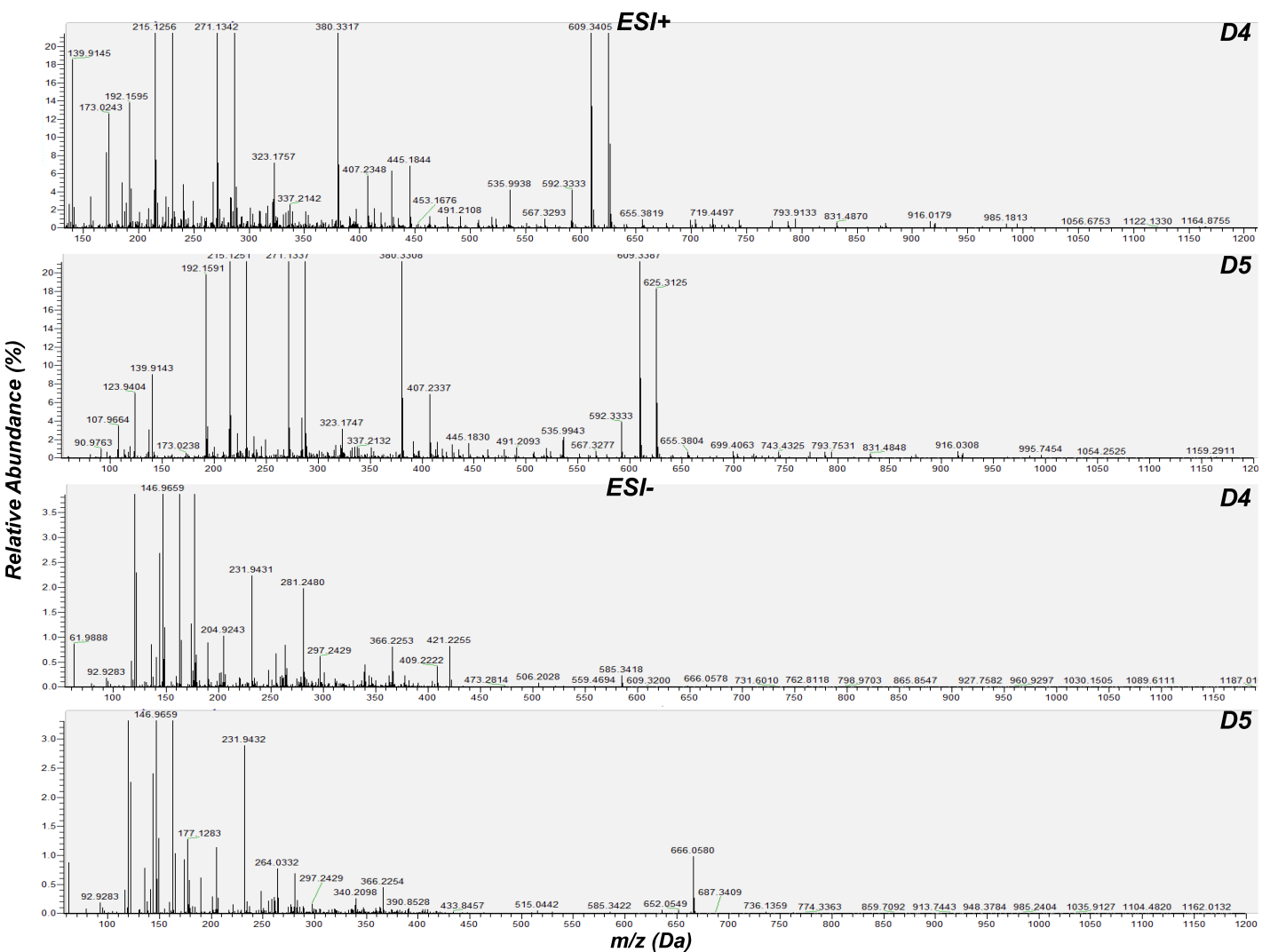


**Supplementary Figure 3 Cumulative mass spectra of *Rhizoctonia solani* pure cultures four (D4) and five (D5) days following treatments.** Data were aquired in positive (ESI^+^) and negative (ESI^-^) electrospray modes performing direct infusion mass spectrometry (DIMS) analysis in the range 50-1200 Da using an LTQ Orbitrap Classic analyzer. The software XCalibur 2.2 was used.

**
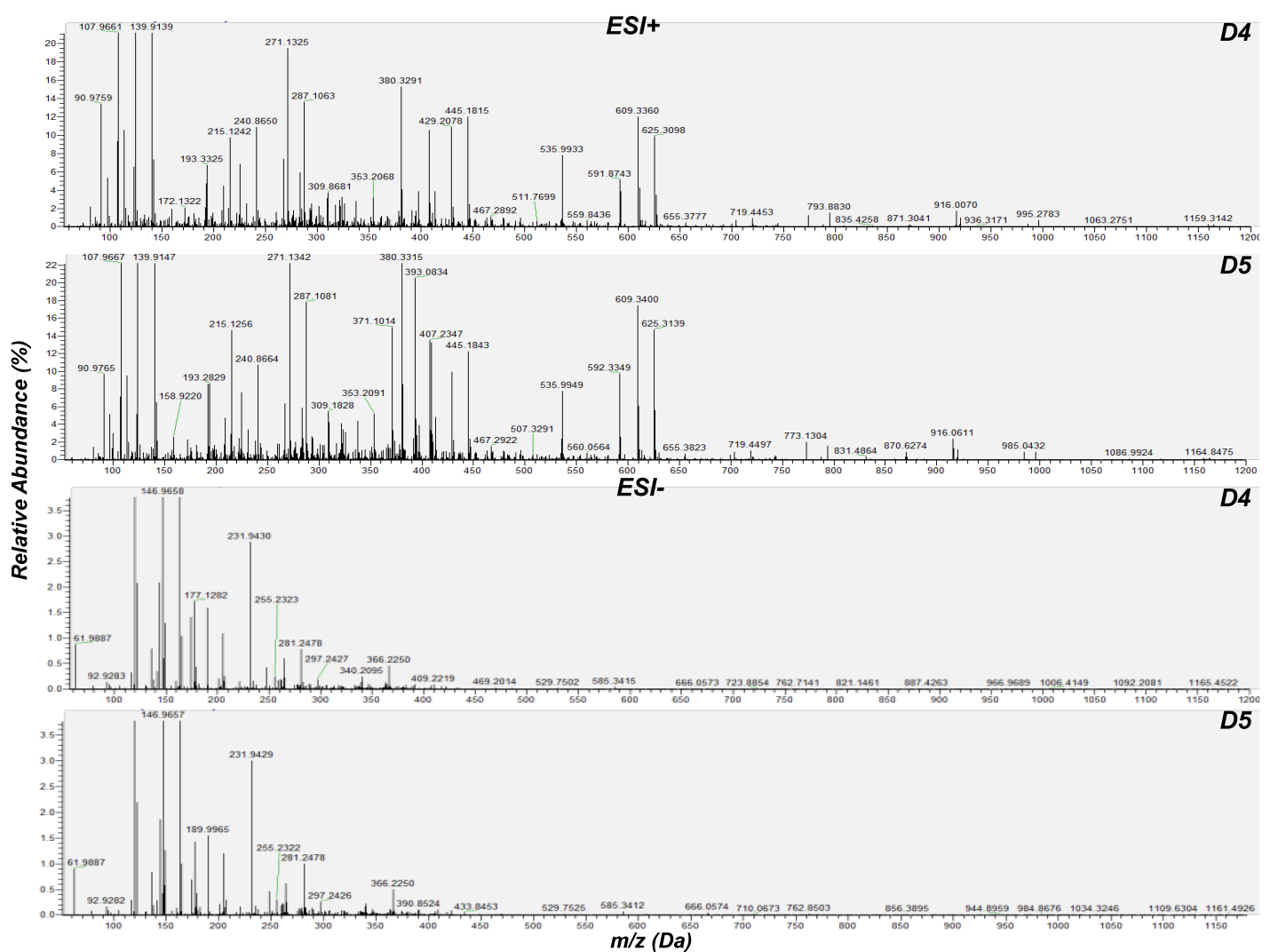
**

**Supplementary Figure 4 Cumulative mass spectra of *Stachybotrys elegans* pure cultures four (D4) and five (D5) days following treatments.** Data were aquired in positive (ESI^+^) and negative (ESI^-^) electrospray modes performing direct infusion mass spectrometry (DIMS) analysis in the range 50-1200 Da. The software XCalibur 2.2 was used.

**
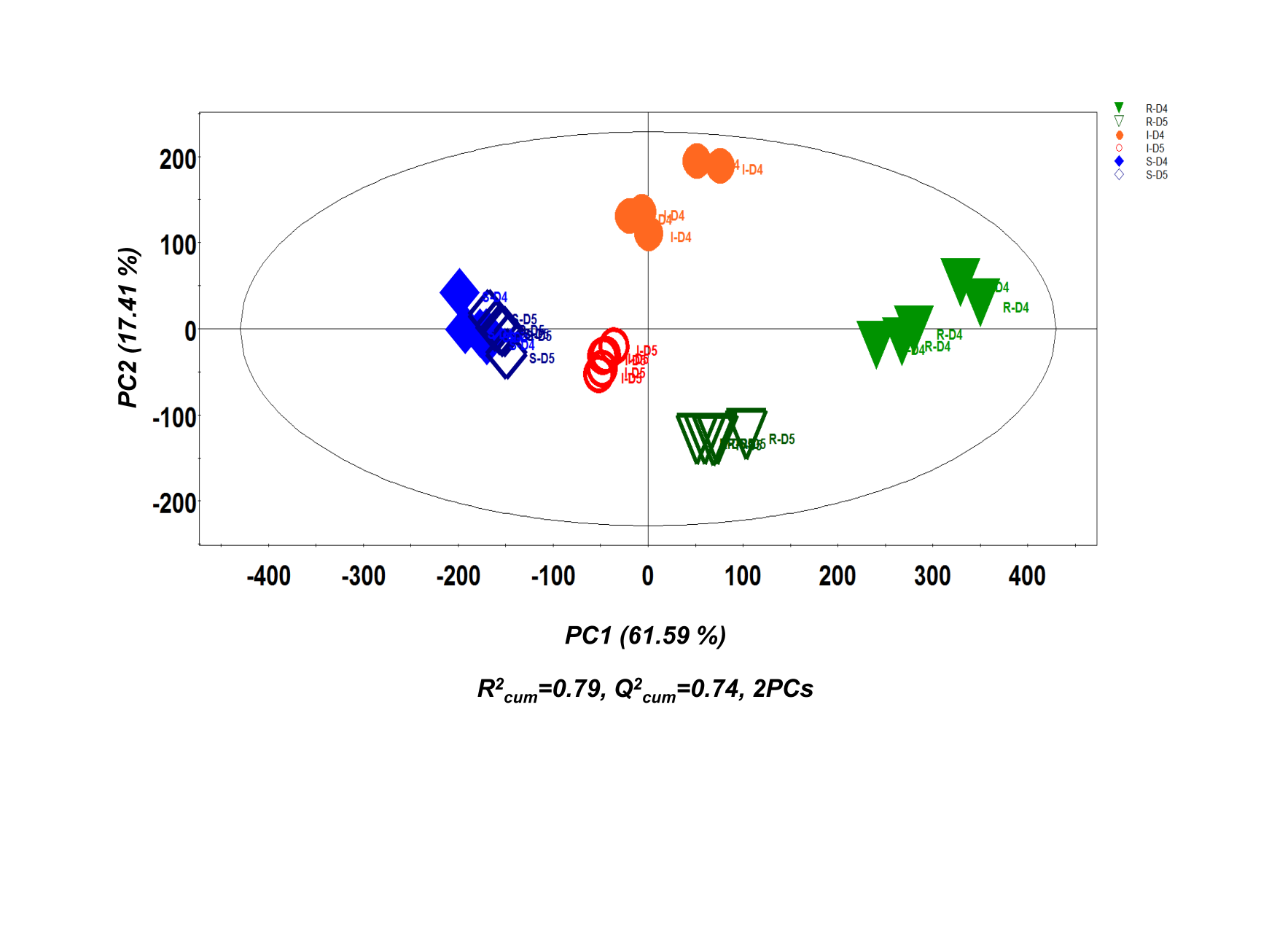
**

**Supplementary Figure 5 Principal components analysis (PCA) PC1/PC2 score plot of profiles of secondary metabolites of *Rhizoctonia solani* (R), *Stachybotrys elegans* (S), and their dual-cultures (I) recorded in positive (ESI^+^) and negative (ESI^-^) electrospray modes four (D4) and five (D5) days following inoculation.** The ellipse represents the Hotelling T2 with 95% confidence interval. Five (5) biological replications were performed per treatment [*Q^2^*; The fraction of the total variation of *X* that can be predicted by the principal components (PC), as estimated by cross-validation; *R^2^*=the fraction of the sum of squares of all *X’*s explained by the current PC].


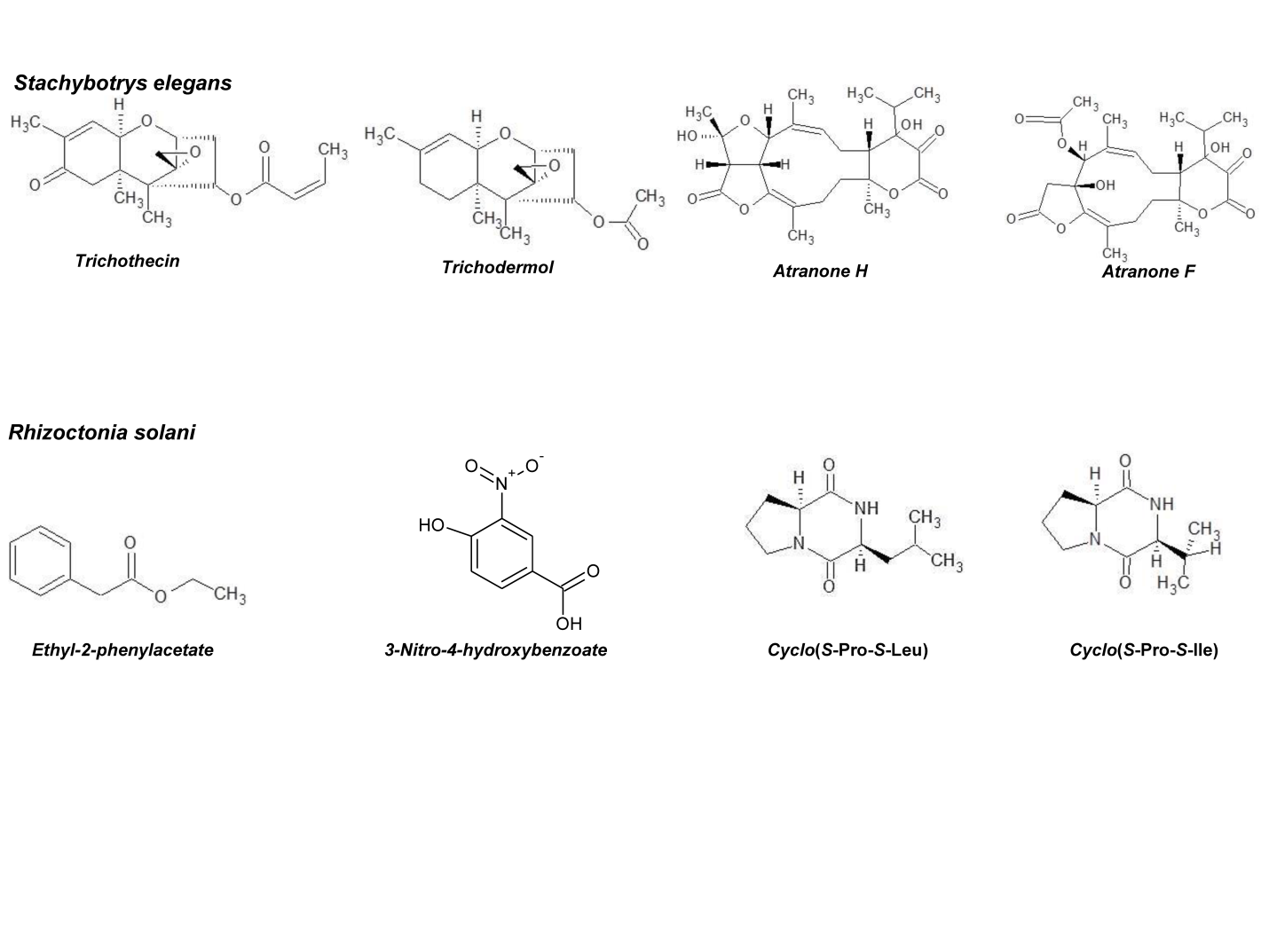


**Supplementary Figure 6 Chemical structures of representative *Stachybotrys elegans* and *Rhizoctonia solani* metabolites induced during their mycoparasitic interaction.**

**
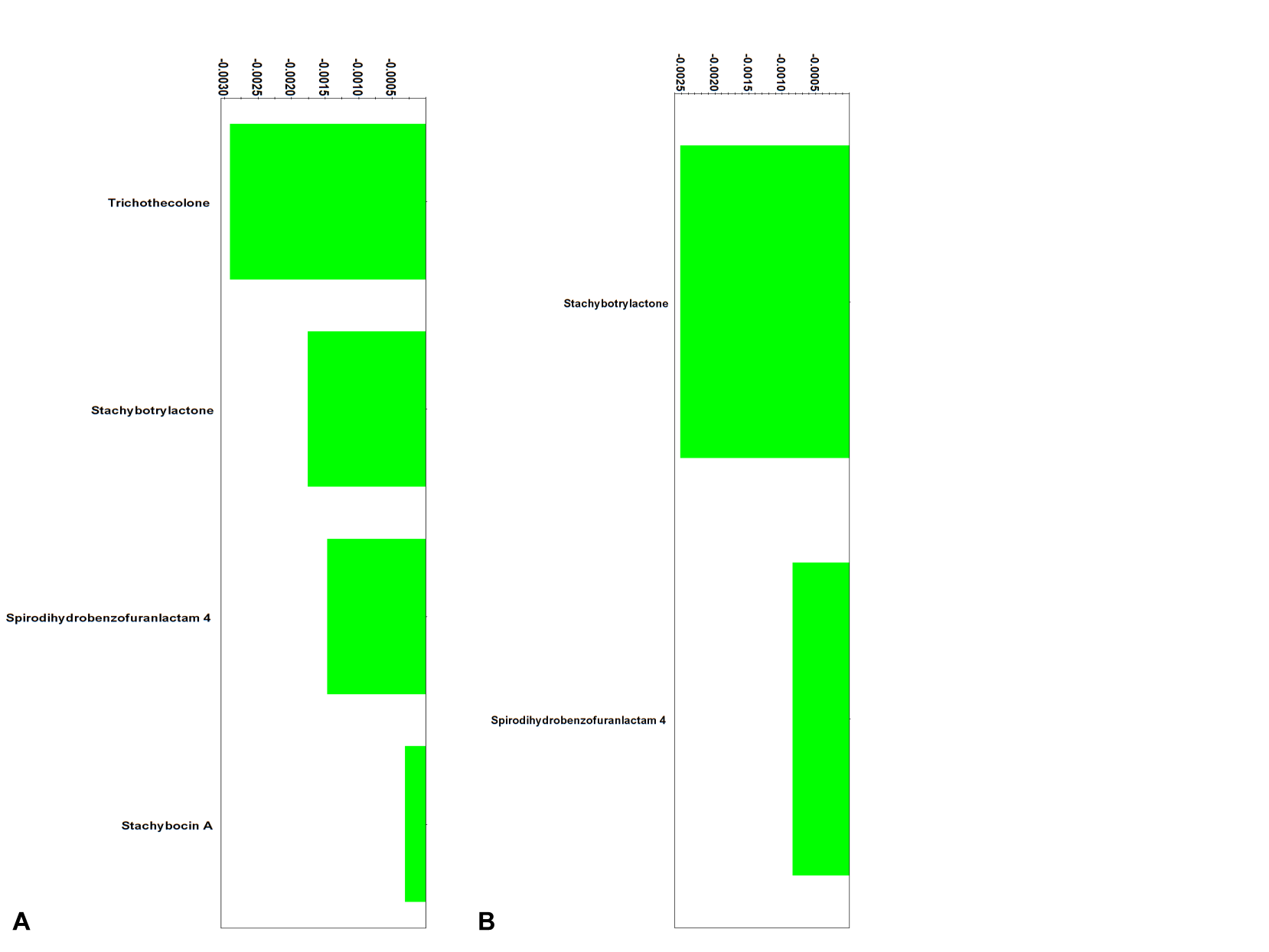
**

**Supplementary Figure 7 Partial least squares (PLS) coefficient plots for the comparison between *Stachybotrys elegans*pure cultures and its dual-cultures with *Rhizoctonia solani* with values of scaled and centered PLS regression coefficients (CoeffCS).** Metabolites present only in pure *Stachybotrys* cultures are displayed.
